# The Impact of Angiotensin-Converting Enzyme 2 (ACE2) Expression on the Incidence and Severity of COVID-19 Infection

**DOI:** 10.3390/pathogens10030379

**Published:** 2021-03-22

**Authors:** Ahmed O. Kaseb, Yehia I. Mohamed, Alexandre E. Malek, Issam I. Raad, Lina Altameemi, Dan Li, Omar A. Kaseb, Safa A. Kaseb, Abdelhafez Selim, Qing Ma

**Affiliations:** 1 Departments of Gastrointestinal Medical Oncology, The University of Texas MD Anderson Cancer Center, Houston, TX 77030, USA; yimohamed@mdanderson.org (Y.I.M.); laltame1@hurleymc.com (L.A.); 2 Infectious Diseases, Infection Control and Employee Health, The University of Texas MD Anderson Cancer Center, Houston, TX 77030, USA; aemalek@mdanderson.org (A.E.M.); iraad@mdanderson.org (I.I.R.); 3 Department of Hematopoietic Biology and Malignancy, The University of Texas MD Anderson Cancer Center, Houston, TX 77030, USA; danli@mdanderson.org (D.L.); qma@mdanderson.org (Q.M.); 4 Iman Academy, Webster, TX 77598, USA; okaseb12@gmail.com (O.A.K.); skaseb80@gmail.com (S.A.K.); 5 Philadelphia College of Osteopathic Medicine (PCOM), Philadelphia, PA 19131, USA; abdu94@yahoo.com

**Keywords:** COVID-19, ACE2, pandemic, coronavirus, SARS-CoV-2

## Abstract

The novel coronavirus disease 2019 (COVID-19) pandemic has led to an unprecedented threat to the international community and raised major concerns in terms of public health safety. Although our current understanding of the complexity of COVID-19 pathogenesis remains limited, the infection is largely mediated by the interaction of viral spike protein and angiotensin-converting enzyme 2 (ACE2). The functional importance of ACE2 in different demographic and comorbid conditions may explain the significant variation in incidence and mortality of COVID-19 in vulnerable groups, and highlights its candidacy as a potential therapeutic target. We provide evidence supporting the idea that differences in incidence and severity of COVID-19 infection may be related to ACE2. Emerging data based on the prevalence and severity of COVID-19 among those with established high levels of ACE2 expression strongly support our hypothesis. Considering the burden of COVID-19 infection in these vulnerable groups and the impact of the potential therapeutic and preventive measures that would result from adopting ACE2-driven anti-viral strategies, our hypothesis may expedite global efforts to control the current COVID-19 pandemic.

## 1. Introduction

The novel coronavirus disease 2019 infection, dubbed as coronavirus disease 2019 (COVID-19), has become a worldwide pandemic with a rapid global spread. The incidence rate and death curve of COVID-19 are noticeably higher in patients of older ages, males, certain ethnicities, and patients with certain comorbidities [1,2,3,4,5,6,7,8,9,10,11]. The circumstances are complicated in both low-income and developed countries: the former lack necessary healthcare basics to combat the pandemic, and the latter struggle with quick containment actions such as implementing contact tracing and developing diagnostic tests with rapid turnaround time. The current situation calls for further efforts in exploring simple and implementation-ready strategies in order to identify and stratify patients who are at the highest risk of contracting COVID-19, and to predict the severity of infection.

**Figure 1:**
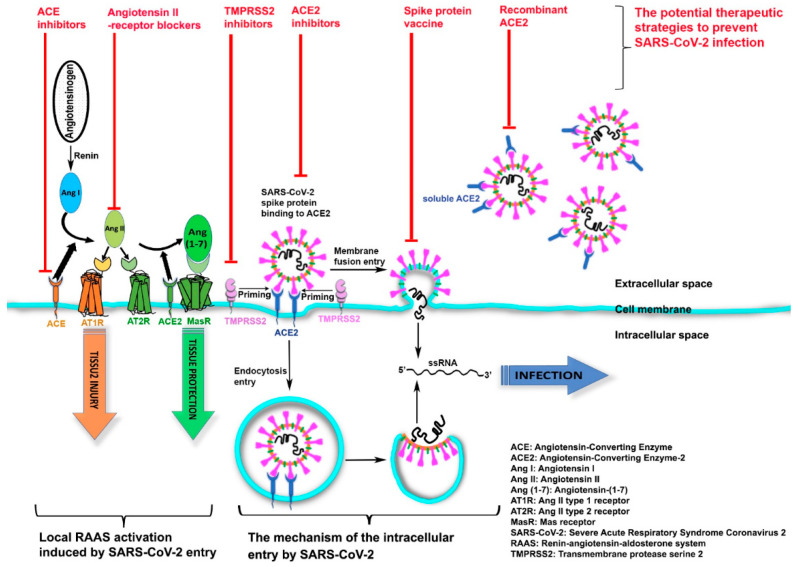
The mechanism of the intracellular entry by SARS-CoV-2 and the potential therapeutic strategies to prevent SARS-CoV-2 infection.

Coronaviruses, including severe acute respiratory syndrome coronavirus 2 (SARS-CoV-2), are single-stranded, ribonucleic acid (RNA), large envelope and positive sense viruses [12]. A recent study demonstrated that SARS-CoV-2 enters human cells by binding to the transmembrane receptor angiotensin-converting enzyme 2 (ACE2) (Figure 1) [13]. We hypothesize that the COVID-19 infection may be related to ACE2 activity. We take the consideration of the severity of the COVID-19 infection in vulnerable groups, and the potential impact of novel anti-viral therapeutics based ACE2 in tackling the virus.

## 2. Search Strategy and Selection Criteria

Data for this review were identified by searches of Medline, PubMed and electronic databases such as Google Scholar; only articles published in English up to March 1st 2021 were included. References from relevant articles using the search terms “COVID-19”, “Coronavirus”, “SARS coronavirus 2”, “ACE2”, and “SARS-CoV-2” were used; retrospective and prospective studies, reviews and gene studies were included.

## 3. Implication of ACE2 Receptor in SARS-CoV-2 Cellular Entry

SARS-CoV-2 host-cell entry is mediated by ACE2 binding to the viral spike protein, whose S1 domain has been primed by transmembrane protease serine 2 (TMPRSS2) [14] (Figure 1). This binding results in SARS-CoV-2 uptake into host-membrane endosomes (via endocytosis) or viral and host plasma membranes fusion (membrane-fusing entry), both of which lead to SARS-CoV-2 entry and host infection [15,16]. Subsequently, the host renin–angiotensin–aldosterone system (RAAS) is activated, which causes tissue injuries including an array of cardiovascular and renal diseases [17]. As part of the RAAS, renin cleaves angiotensinogen and generates angiotensin I (Ang I) which anchors on the target-cell membrane via ACE—the angiotensin-converting enzyme that opposes the actions of ACE2. After further cleavage by ACE, Ang I forms angiotensin II (Ang II)-the peptide which binds to and activates the Ang II type 1 receptor (AT1R), thus promoting severe tissue injury. In contrast, ACE2, another transmembrane enzyme, inactivates Ang II by removing the carboxyterminal amino acid of Ang II and producing angiotensin-(1-7) (Ang-(1-7)), a heptapeptide with a potent vasodilator function. Ang-(1-7), via activation of its G protein-coupled Mas receptor (MasR), negatively regulates RAAS and leads to the final step in opposing host tissue injury.

## 4. Variation of ACE2 Receptor as a Function of Age, Gender, Ethnicity, and COVID-19 Severity

Rapidly evolving data indicate that men, the elderly, individuals with multiple comorbidities, and certain ethnic groups are at increased risk of developing and dying from the COVID-19 infection. The severity of COVID-19 infection, complications and high mortality rate are related to advanced age [6,18,19]. It is noteworthy that the expression of ACE2 increases with age and might correlate with a high risk of acquiring COVID-19 pneumonia, and severe outcomes. Kelvin To et al. have recently reported the association between age and COVID-19 high viral load among a cohort of 23 patients; high viral load was correlated with COVID-19 severity and associated with high ACE2 expression in this age group of older patients [20]. A recent study assessed the ACE2 gene expression in the nasal cavity of 305 patients aged 4–60 years old; children (4–9 years old, n = 45) showed lower ACE2 expression in the nasal epithelium than other age groups, with a significantly higher ACE2 expression with advancing age [21].

The differences of COVID-19 severity and mortality between males and females have been established; males with COVID-19 are at greater risk of complications and death [5,22]. Published data indicate that the correlation between gender and severity of COVID-19 in males may be related to higher ACE2 expression levels [23,24,25,26]. The distribution of ACE2 expression is more widespread in males than females, that could explain the gender differences in COVID-19 infection and severity [27]. In a cohort of 65 patients with clinical suspicion of COVID19, 60% were male and the median age was 56 years old. Among the confirmed COVID-19 patients, 64.3% were male and the median age was 56 years old (46.25–67.8), and among those who were COVID-19 negative, 33.3% were male and the median age was 50 years old (35.5–70.5) [28].

The World Health Organization (WHO) reported that there were 93,706,818 confirmed COVID-19 cases by January 2021, with cases outside China being mainly in the Americas, Europe, and Southeast Asia, with fewer cases in Africa (WHO, 2021); this indicates that ethnicity might play a role in the susceptibility and severity of COVID-19 infection. Black and Asian ethnic populations have been reported to have a higher risk of COVID-19 infection than Caucasians; higher ACE2 expression was observed in Asians than in American and European populations and this could explain the differences in distribution for Asians and Caucasians [29]. Nevertheless, other studies have shown similar ACE2 expression levels in different races and ethnicities; ACE2 expression and severity of COVID-19 remains uncertain [30,31,32].

## 5. ACE2 Associations with Cardiovascular Disease, Hypertension, and COVID-19 Infection

ACE2 is widely expressed in the adult human heart and single cell analysis has revealed high expression of ACE2 in pericytes [33]. In addition, ACE2 expression regulates blood pressure and plays an important role in the pathophysiology of hypertension [34]. Published data have shown that membrane-bound ACE2 expression is important to prevent the development and progression of cardiovascular disease (CVD) [35]. However, increased circulating ACE2 in plasma and urine is a biomarker, and associated with high risk of CVD [36]. In patients suffering from CVD, the ACE2 internalization and the loss of cell-surface ACE2 following COVID-19 infection could lead to worsening of their preexisting CVD. ACE2 loss in the tubular epithelium of the kidney could increase blood volume and pressure due to altered sodium transport [13]. Hypokalemia and uncontrolled blood pressure have been commonly described in patients with a more severe COVID-19 infection which confirms the stimulating role of the Ang II–AT1 receptor axis [37]. Studies showed that recombinant human ACE2 (rhACE2) infusion could produce high levels of angiotensin-(1-7) and prevent cardiovascular injury induced by angiotensin-II [38]. However, this could also lead to hypotension in COVID-19 patients in later stages of the disease [13]. There is no evidence to support the claim that angiotensin converting enzyme inhibitors (ACEIs)/angiotensin II receptor blockers (ARBs) upregulate expression of ACE2 in the human lung. While some studies have shown that ACEIs/ARBS can lower mortality in hypertensive patients with COVID-19 infection, conversely, other studies suggest no protective value against COVID-19 [39].

## 6. ACE2 Associations with COVID-19 Infection and Lung Function

ACE2 gene expression levels are increased in the airway epithelium of smokers as well as in chronic obstructive pulmonary disease (COPD) patients. These patients have a higher risk for COVID-19 infection [40]. Following COVID-19 infection, Ang II activity plays a major role in contributing to multiple organ injuries [41,42]; previous studies have especially shown that the Ang II level increases concurrently with local RAAS activation following downregulation of ACE2 in the lung [43,44,45]. In a small study evaluating 12 COVID-19 patients, the elevated levels of Ang II were correlated with severe lung injury and higher viral load [42]. In an experimental study on mice, recombinant ACE2 infusion could restore ACE2 level and reverse the process of lung injury [46,47]. In a phase 2 trial assessing the acute respiratory distress syndrome in humans, administrating recombinant ACE2 also safely reduced Ang II levels [48]. These observations have prompted a trial to examine the role of the recombinant ACE2 protein in controlling the RAAS and protecting against organ injury (NCT04287686). COVID-19 treatment trials using the Ang II receptor antagonist losartan are being conducted among hospitalized (NCT04312009) and non-hospitalized (NCT04311177) patients who have not previously received RAAS-inhibitor treatment.

## 7. ACE2 Expression in Nasopharyngeal and Oropharyngeal Swabs of COVID-19 Patients

COVID-19 infection symptoms are mostly respiratory, and the main method of transmission of infection is through liquid droplets coming out of nasal and oral cavities [49], with evidence showing that ACE2 is the receptor responsible for COVID-19 entry [50]. A recent study confirmed the overexpression of ACE2 in nasal and oral cavities of COVID-19 patients. Nasopharyngeal and oropharyngeal swabs from 63 suspected COVID-19 cases were collected and analyzed; ACE2 expression level was used to identify COVID-19 positive patients from negative subjects, suggesting its utility as a biomarker for COVID19 detection [51].

## 8. ACE2 Expression in the Central Nervous System

COVID-19 affects various tissues and body organs other than just the lungs, including the central nervous system; neurological symptoms were evident in a cohort of 214 COVID-19 positive patients, where 36.4% of them presented with neurological symptoms such as hyposmia, hypgeusia, and stroke, as well as less specific symptoms such as dizziness, headache, and seizures [52]. These symptoms could be used for preliminary screening of a patient’s condition. A recent study demonstrated that ACE2 is highly expressed in astrocytes of subtantia nigra, cortex and oligodendrocyte precursor cells, while hippocampus cells had low ACE2 expression levels, with an ACE2 expression level of zero in the cerebellum, spinal cord, and neuronal epithelium [53]. The data suggest that the brain is a high-risk organ and could provide a possible mechanism for the neurological symptoms in COVID-19 patients.

## 9. ACE2 Expression and Gastrointestinal Tract

Gastrointestinal symptoms are common in patients with COVID-19 and have been described earlier in the disease [54,55]. A high level of ACE2 expression was found in the human gastrointestinal tract [56]. As ACE2 plays a crucial role in the cellular entry of SARS-CoV-2, the variation of ACE2 expression might affect the severity of infection. Another interesting aspect is the potential interaction between ACE2 expression and gut microbiota in the severity of the COVID-19 infection. A published study showed that ACE2 has the ability to modulate gut microbiota and confer beneficial effects on the cardiopulmonary health [57].

## 10. ACE2 Expression and Obesity

More recently, a high prevalence of obesity among patients with severe COVID-19 has been reported, and these patients require intensive care admissions and mechanical ventilations [58]. This could be explained by an imbalance in the RAAS system secondary to obesity [59], and possibly linked to robust systemic response secondary to SARS-CoV-2 infection. Recent meta-analysis showed that individuals with lower level of ACE2 expression in adipose tissue are more likely to be associated with type 2 diabetes mellitus, cardiovascular comorbidities, metabolic syndrome, and a higher body mass index, which are all risk factors of severe COVID-19 [60].

## 11. ACE2 and ADAM17 Expression in Cancer Patients with COVID-19

Little is known about the difference in the expression of ACE2 between normal and tumor cells. Interestingly, a study conducted by Xu et al. showed that the ACE2-positive rate in tumor cells was remarkably higher compared to normal host cells [61]. This may partially explain why cancer patients with COVID-19 are more prone to developing infections. On the other hand, the serine protease ADAM metallopeptidase domain 17 (ADAM17) is responsible for the ACE2 cleavage and the release of its ectodomain [62]. The soluble ACE2 binds to SARS-CoV-2, and may serve as viral decoy substrate to prevent the virus from host cell entry and internalization. In cancer patients, it is unclear whether the expression or the activity of ADAM17 is increased. Some anti-cancer therapies might inhibit the activity of ADAM17, which in theory would decrease the release of soluble ACE2 in the circulation or extracellular compartment and subsequently enhance the internalization of SARS-CoV-2 into the cells.

## 12. Genetic Polymorphism of ACE2 Gene and SARS-CoV-2 Spike Protein Mutations

Given the vital interaction between the ACE2 receptor and the SARS-CoV-2 spike protein, and its implication for susceptibility and infection, we argue that ACE2 genetic variation or mutations could carry a different clinical outcome and change the severity of disease. Although two studies evaluating the impact of ACE2 variants showed no strong correlation or influence on COVID-19 severity, larger studies are imperative to further understand the complexity of gene variant expression and its effect on the prognosis of COVID-19. On the other hand, a SARS-CoV-2 variant with spike protein D614G mutation has been demonstrated to enhance viral infectivity [63,64,65]. Interestingly, recent data demonstrate that interferons (IFNs) and SARS-CoV-2 can induce the expression of a truncated, not full-length ACE2 isoform with unknown function [66,67,68]. This truncated ACE2 isoform lacks high-affinity binding domains required for SARS-CoV-2 spike glycoprotein, and thus is unlikely to mediate virus entry and enhance risk of infection.

## 13. Discussion

The current review provides evidence to substantiate the hypothesis about the link between ACE2 expression and strong risk factors for disease severity and mortality such as advanced age, male gender, black and asian ethnicities. Additionally, ACE2 expression is also associated with certain comorbidities that contribute to the risk of severe COVID-19 infection and mortality including: cardiovascular disease, hypertension, chronic lung conditions such as COPD, and obesity. Needless to say, the current COVID-19 pandemic calls for innovation in preventive and therapeutic approaches, as well as risk stratification to identify at-risk populations and vulnerable groups who may need special attention (Table 1).

The current review supports our hypothesis that differences in incidence and severity of COVID-19 infection might be directly related to the degree of ACE2 expression, which could have meaningful implications for future directions in developing a global strategy to combat risks of severe infection and mortality. For example, after prospective validation, measuring blood levels of ACE2 may provide a risk-stratification opportunity to identify individuals at greater risk of infection and for severe illness. Furthermore, targeting the ACE2 system may provide a novel approach to protect against COVID-19 infection, and, for individual patient, this may aid in monitoring responses to preventive measures and treatment interventions. Thus, focusing on the potential therapeutic strategy enabled by targeting ACE2 is especially important. Notably, SARS-CoV-2 internalizes ACE2 upon entering the target cell, that could potentially reduce cell-surface ACE2 levels, and cause unopposed Ang II accumulation, Ang-(1-7) downregulation, and RAAS activation. Potential therapeutic strategies to prevent SARS-CoV-2 infection include RAAS inhibitors (such as ACE inhibitors and Ang II-receptor blockers) modifying ACE2 levels or activity in target cells, a vaccine targeting the spike protein of SARS-CoV-2, TMPRSS2 inhibitors blocking spike-protein priming, and soluble recombinant ACE2 as a virus trap and inactivator that could competitively bind with SARS-CoV-2 to slow viral entry into target cells (Figure 1).

Major efforts are under way to develop novel effective therapeutics for COVID-19, but the validation of new potential agents may take a long time. Therefore, applying the drug repurposing strategy to identify antiviral effects against SARS-CoV-2 has been a very active area [69], involving the use of existing drugs that have been already approved for use in clinical settings or trials. In addition, because drug safety has been substantiated in those trials, drug administration can be timely provided to treat COVID-19. [70] The current model shows that the spike protein of COVID-19, in complex with human ACE2, facilitates infection and suggests that disruption of the interface of viral spike protein and ACE2 is a reasonable strategy for structure-based drug discovery [71]. Several three-dimensional models for this protein–protein complex have been produced [72,73,74,75]. The in silico structure-based virtual screening approach has been used for drug repositioning purposes [76,77]. However, the drugs suggested by these studies require further assessment in vitro, followed by pre-clinical and clinical studies. Animal models of ACE2 have been utilized in the mechanistic research and preclinical studies of SARS-CoV-2 infection and COVID-19 drug development. Several human ACE2 transgenic mouse models are available with the ubiquitous, tissue-specific, or endogenous mouse ACE2 promoter to control gene expression. These mice are susceptible to SARS-CoV-2 infection, and subsequently develop various ranges of symptomatic COVID-19 disease [78,79,80,81]. The data suggest that human ACE2 transgenic mouse models are not only useful for understanding SARS-CoV-2 transmission and pathogenicity, but also essential tools to evaluate potential COVID-19 therapeutics [82].

## 14. Conclusions

The current pandemic situation highlights the critical importance of developing simple risk-stratification and therapeutic approaches that are ready to be implemented in both high- and low-resource settings, in order to pinpoint populations at the highest risk of contracting COVID-19 and allocate resources to vulnerable, at-risk patients. Furthermore, optimal strategies should be able to provide prevention and treatment in low-resource and low-income areas, thus reducing economic and mental stress in the context of currently exhausted healthcare systems.

## Figures and Tables

**Table 1:** Correlations of demographics, age, gender, race, and comorbidities with COVID-19 risk and infectivity.

Variable	Study	Year	Number of Patients	Type of Study
Race and COVID-19	The immune dysregulations in COVID-19: implications for the management of rheumatic diseases. Modern Rheumatology	2020		Systematic review
	Asians Do Not Exhibit Elevated Expression or Unique Genetic Polymorphisms for ACE2, the Cell-Entry Receptor of SARS-CoV-2	2020		Gene
	Tobacco-use disparity in gene expression of ACE2, the receptor of 2019-nCov	2020		Gene
	The impact of ethnicity on clinical outcomes in COVID-19: A systematic review	2020		Systematic review
Age and COVID-19	Association between age and clinical characteristics and outcomes of COVID-19	2020	221	Retrospective
	Estimates of the severity of coronavirus disease 2019: a model-based analysis	2020	189	Retrospective
	Clinical course and risk factors for mortality of adult inpatients with COVID-19 in Wuhan, China: a retrospective cohort study	2020	191	Retrospective
	Temporal profiles of viral load in posterior oropharyngeal saliva samples and serum antibody responses during infection by SARS-CoV-2: an observational cohort study	2020	23	Pprospective
	Detection of SARS-CoV-2 antibodies is insufficient for the diagnosis of active or cured COVID-19	2020	65	Retrospective
	Nasal Gene Expression of Angiotensin-Converting Enzyme 2 in Children and Adults	2020	305	Retrospective
Gender and COVID-19	Angiotensin-converting enzyme 2 (ACE2), SARS-CoV-2 and the pathophysiology of coronavirus disease 2019	2020		Systematic review
	Gender Differences in Patients With COVID-19: Focus on Severity and Mortality	2020	1623	Retrospective
	Clinical course and outcomes of critically ill patients with SARS-CoV-2 pneumonia in Wuhan, China: a single-centered, retrospective, observational study	2020	52	Retrospective
	Clinical Characteristics of Coronavirus Disease 2019 in China	2020	1099	Retrospective
	Sex differences in COVID-19: candidate pathways, genetics of ACE2, and sex hormones	2021		Systematic review
	ACE2: the molecular doorway to SARS-CoV-2. Cell & Bioscience	2020		Systematic review
	Molecular Mechanisms Lead to Sex-Specific COVID-19 Prognosis and Targeted Therapies	2020		Systematic review
ACE2, HTN, CVD, and COVID-19	Hypokalemia and clinical implications in patients with coronavirus disease 2019 (COVID-19)	2020	175	Clinical, retrospective
	COVID-19, ACE2, and the cardiovascular consequences	2020		Systematic review
	Cardiovascular Implications of Fatal Outcomes of Patients With Coronavirus Disease 2019 (COVID-19)	2020	187	Retrospective
	Positive association of angiotensin II receptor blockers, not angiotensin-converting enzyme inhibitors, with an increased vulnerability to SARS-CoV-2 infection in patients hospitalized for suspected COVID-19 pneumonia	2020	684	Retrospective
ACE2, lung function, and COVID-19	Smoking Upregulates Angiotensin-Converting Enzyme-2 Receptor: A Potential Adhesion Site for Novel Coronavirus SARS-CoV-2 (Covid-19)	2020		Systematic review
	Angiotensin receptor blockers as tentative SARS-CoV-2 therapeutics	2020		Systematic review
	Clinical and biochemical indexes from 2019-nCoV infected patients linked to viral loads and lung injury	2020	12	Retrospective
ACE2 expression and CNS	How Does SARS-CoV-2 Affect the Central Nervous System? A Working Hypothesis	2020		Review
	The scRNA-seq expression profiling of the receptor ACE2 and the cellular protease TMPRSS2 reveals human organs susceptible to COVID-19 infection	2020		Gene
ACE2 expression in nasopharyngeal and oropharyngeal swabs of COVID-19 patients	SARS-CoV-2, ACE2 expression, and systemic organ invasion	2021		Review
	ACE2: Evidence of role as entry receptor for SARS-CoV-2 and implications in comorbidities	2020		Review
	Expression profiles of the SARS-CoV-2 host invasion genes in nasopharyngeal and oropharyngeal swabs of COVID-19 patients	2020	63	Gene
ACE2 expression and gastrointestinal tract	Quantitative mRNA expression profiling of ACE 2, a novel homologue of angiotensin converting enzyme	2002		Gene
	ACE2 and Microbiota: Emerging Targets for Cardiopulmonary Disease Therapy	2015		Review

Abbreviations: ACE, angiotensin converting enzyme; COVID-19, coronavirus disease 2019; ARDS, adult respiratory distress syndrome.

## Data Availability

Not applicable.

## Abbreviations

ACE2	Angiotensin-converting enzyme 2
ACEI	Angiotensin converting enzyme inhibitor
ADAM17	ADAM metallopeptidase domain 17
Ang I	Angiotensin I
Ang II	Angiotensin II
ARBs	Angiotensin II receptor blockers
COPD	Chronic obstructive pulmonary disease
COVID-19	Coronavirus disease 2019
CVD	Cardiovascular disease
eQTLs	Expression quantitative loci
MasR	Mas receptor
RAAS	Renin–angiotensin–aldosterone system
RAS	Renin–angiotensin system
rhACCE2	Recombinant human ACE2
RNA	Ribonucleic acid
SARS-CoV-2	Severe acute respiratory syndrome coronavirus 2
scRNA	Single-cell RNA
TMPRSS2	Transmembrane protease serine 2
